# What is the prevalence of self-harming and suicidal behaviour in under 18s with autism spectrum disorder, with or without an intellectual disability?

**DOI:** 10.1192/bjo.2021.743

**Published:** 2021-06-18

**Authors:** Rosalind Oliphant, Eleanor Smith, Victoria Grahame

**Affiliations:** Cumbria, Northumberland, Tyne And Wear NHS Foundation Trust

## Abstract

**Aims:**

The aims of this systematic review are to summarise data on the prevalence of suicidal behaviours and self-harm in under 18s with Autism Spectrum Disorder (ASD) and consider the impact of Intellectual Disability (ID). It was hypothesised that the prevalence of these behaviours may be higher in under 18s with ASD than in the general population.

**Background:**

In the general population, rates of self-harm and suicide in under 18s are of increasing concern. Whilst there is an emerging evidence base considering suicidality in autistic adults, less in known about the experience of under 18s. There has been very little research focused on how self-harm seen within the general population presents in the context of ASD and whether it continues to be a predictor of future suicidal behaviour. This may be partly due to self-harm being considered alongside Self-Injurious Behaviours (SIB), which have long been recognised as part of the clinical presentation of ASD and may have other functions (e.g. fulfilling sensory stimulation needs).

**Method:**

A systematic literature search was conducted in line with PRISMA guidelines. For this review, all papers that included data on prevalence of self-harm and/or suicidal behaviours in under 18s with ASD were included. Studies that only reported on the prevalence of the broader entity of SIB (characterised as stereotypic or habitual) were excluded.

**Result:**

338 papers were initially identified and 9 met eligibility criteria. There was considerable variation in how different aspects of self-harm and suicidal behaviours were addressed between groups and also between population samples, making it difficult to generalise the findings. The prevalence of self-harming and suicidal behaviours ranged from 7% to 73%, indicating that this is a clinically significant problem for this patient group. The only study that considered the impact of co-existing ID did not identify significant differences between groups (ID vs no ID).

**Conclusion:**

There was variation in the reported prevalence rates but results suggested that rates of both self-harm and suicidal behaviour may be elevated in under 18s with ASD compared to the general population. This is in keeping with literature relating to autistic adults but in contrast to conclusions of a previous systematic review. This review highlights the need for further research to explore the experience of self-harm and suicidal behaviour in autistic children and young people.

